# A protocol for an interventional study on the impact of transcutaneous parasacral nerve stimulation in children with functional constipation

**DOI:** 10.1097/MD.0000000000023745

**Published:** 2020-12-18

**Authors:** Giovanna Maria Coelho, Nilton Carlos Machado, Mary de Assis Carvalho, Rebeca Mayara Padilha Rego, Stefani Rabelo Vieira, Erika Veruska Paiva Ortolan, Pedro Luiz Toledo de Arruda Lourenção

**Affiliations:** a Botucatu Medical School, São Paulo State University (UNESP); b Department of Pediatrics, Division of Pediatric Gastroenterology, Hepatology and Nutrition Botucatu Medical School, São Paulo State University (UNESP), São Paulo; c Department of Surgery and Orthopedics - Division of Pediatric Surgery, Botucatu Medical School, São Paulo State University (UNESP), São Paulo, Brazil.

**Keywords:** child, constipation, neurostimulation

## Abstract

**Introduction:**

Transcutaneous parasacral nerve stimulation (TPNS) via electrodes placed over the sacrum can activate afferent neuronal networks noninvasively, leading to sacral reflexes that may improve colonic motility. Thus, TPNS can be considered a promising, noninvasive, and safe method for the treatment of constipation. However, there is no published study investigating its use in children with functional constipation. This is a single-center, prospective, longitudinal, and interventional study designed to assess the applicability and clinical outcomes of TPNS in functionally constipated children.

**Patient concerns:**

Parents or guardians of patients will be informed of the purpose of the study and will sign an informed consent form. The participants may leave the study at any time without any restrictions.

**Diagnosis:**

Twenty-eight children (7–18 years old) who were diagnosed with intestinal constipation (Rome IV criteria) will be included.

**Interventions:**

The patients will be submitted to daily sessions of TPNS for a period of 4 or 8 weeks and will be invited to participate in semistructured interviews at 3 or 4 moments: 1 week before the beginning of TPNS; immediately after the 4 and/or 8 weeks of TPNS; and 4 weeks after the end of the intervention period. In these appointments, the aspects related to bowel habits and quality of life will be assessed.

**Outcomes:**

This study will evaluate the increase in the number of bowel movements and stool consistency, the decrease in the number of episodes of retentive fecal incontinence, and the indirect improvement in the overall quality of life.

**Conclusion:**

we expect that this study protocol can show the efficacy of this promising method to assist the treatment of children with functional constipation.

## Abstract 2

Supplemental Digital Content is available in the text

## 1 Introduction

Functional constipation (FC) is considered a common childhood disease worldwide.^[1]^ It accounts for 3% to 5% of pediatrician visits and 10% to 25% referrals to gastroenterologists.^[2]^ Constipation can be commonly associated with infrequent and/or painful defecation, fecal incontinence, and abdominal pain. It is a cause of psychological stress for the child and family, and it has a significant impact on health care costs.^[1]^

The pathophysiology of constipation in children is multifactorial and is associated with many risk factors and their interactions. Several organic diseases cause constipation. However, in most children presenting with constipation, no underlying causal disease can be found. Thus, over 90% of children with this condition have FC.^[1,3]^ At present, the most commonly used diagnostic criteria for FC are the Rome IV definitions (2016), including clinical signs and symptoms that must be present for at least 1 month.^[4,5]^ A vast majority of children with FC respond to standard medical treatment, including pharmacological and nonpharmacological interventions. Pharmacological treatment with laxatives involves 3 steps: disimpactation, maintenance treatment, and weaning. Nonpharmacological interventions involve education, demystification, guidance for toilet training, scheduled toilet seats, establishing a reward system, and keeping a defecation diary.^[1,6,7]^ However, some children may be refractory to these conventional treatments.^[1,2]^

Over the past 2 decades, neurostimulation has been incorporated in clinical practice to treat a variety of problems, ranging from nausea and vomiting to bowel and urinary disorders.^[8–12]^ Neurostimulation gathers a set of procedures involving the circulation of electric current to obtain physiological responses, depending on its intensity, frequency, and pulse width.^[11]^ Various neurostimulation methods have been introduced to treat both bladder and bowel elimination disorders that are resistant to standard therapy.^[13,14]^

Stimulation of spinal sacral nerves via implantable neuromodulatory therapies such as sacral nerve stimulation (SNS) may improve symptoms of constipation. SNS has been increasingly indicated for severe cases of constipation in adults and children.^[15–17]^ However, SNS is an expensive and invasive method that requires at least 1 general anesthetic with complication rates ranging from 17.2% to 50%.^[13,18,19]^

Transcutaneous electrical nerve stimulation (TENS) may be a promising noninvasive and less expensive alternative to SNS.^[14]^ Transcutaneous stimulation can activate afferent neuronal networks noninvasively, leading to sacral reflexes that may improve colonic motility.^[18]^ A systematic review evaluated the effectiveness and safety of TENS to improve constipation in children.^[20]^ The authors concluded that there is not enough evidence to establish conclusions and suggested that randomized controlled trials assessing TENS for the management of childhood constipation should be conducted, addressing methodology and outcomes.^[20]^

Transcutaneous parasacral nerve stimulation (TPNS) via electrodes placed over the sacrum has been applied to reduce bladder contractility and symptoms in children with overactive bladder.^[21–23]^ Multiple studies have also shown that TPNS is useful for the improvement of fecal incontinence in adults.^[24–26]^ However, its use for the treatment of constipation has been minor investigated. Kim et al^[27]^ found significant improvements in constipation symptoms and the number of bowel movements in a group of 15 adults treated with TPNS. On the other hand, Iqbal et al^[18]^ failed to identify a significant improvement of constipation symptoms in 16 patients submitted to TPNS over 4 weeks for 12 hours a day.

In children, the impact of TPNS on constipation symptoms was assessed only in patients who had a variety of associated underlying causes. Veiga et al^[28]^ found a significant reduction in constipation symptoms in 14 children with lower urinary tract dysfunction after 20 sections of TPNS. In another study, the same group also showed resolution of constipation, according to Rome III criteria, in 15 of 25 children with overactive bladder submitted to TPNS.^[29]^ Martins-Moura et al^[30]^ evaluated 18 patients who had developed constipation on the postoperative evolution of a pull-through procedure for Hirschsprung disease or posterior sagittal approach for anorectal malformation. The authors found remission or improvement of constipation in 15 (83.3%) of these patients after TPNS.

Therefore, TPNS is a minimally invasive, well-tolerated, and accessible technique with the potential to improve constipation in the pediatric population. Thus, we decided to assess the applicability and clinical outcomes of TPNS in children with FC.

## 2 Methods

### 2.1 Study design and setting

This is a single-center, prospective, longitudinal, and interventional study designed to evaluate the applicability and clinical outcomes of TPNS in children with FC.

This research will be conducted at the Botucatu Medical School, Sao Paulo State University (UNESP), São Paulo, Brazil. The data collection period will be 18 months long, starting in November 2020. Patients will participate in daily sessions of TPNS for a period of 4 or 8 weeks. All patients will also be invited to participate in semi structured interviews at 3 or 4 moments: 1 week before the start of the intervention; immediately after the 4 weeks of intervention or immediately after the 4 and 8 weeks of intervention; and 4 weeks after the end of the intervention period. In these appointments, the aspects related to bowel habits and quality of life will be evaluated.

### 2.2 Ethics approval and consent

This study will be conducted according to the principles of the Declaration of Helsinki, ISO14155, Data Protection Act, and the Guidelines for Good Clinical Practice. The Research Ethics Committee (REC) of the Botucatu Medical School, UNESP, São Paulo, Brazil, approved this study, registered under number 13760619.4.0000.5411 (see REC, Supplemental Digital Content 1). Parents or guardians of patients will be informed of the purpose of the study and will sign an informed consent form (see informed consent form, Supplemental Digital Content 2). Patients aged 11 to 18 years will sign the respective specific consent form (see specific consent form, Supplemental Digital Content 3). REC will receive all data at the end of the study. The participants may leave the study at any time without any restrictions.

The Brazilian Registry of Clinical Trials (Rebec) identifier for this study is RBR-52f6h2, obtained on July 6, 2020 (UTN number: U1111-1250-1769) and is available at http://www.ensaiosclinicos.gov.br/rg/RBR-52f6h2/.

### 2.3 Patient selection and recruitment

The estimated sample size was 28 patients, calculated for a paired *t*-test (before and after intervention), based on an estimated mean increase of 18 defecations per month (according to data from a previous TPNS study 27), an estimated standard deviation of changes = 30, a 2-tailed α = 0.05 and a test power β= 0.85. The sample will include the Pediatric Gastroenterology Outpatient and Pediatric Surgery Outpatient Clinics of the Botucatu Medical School (UNESP). Patients will be invited to participate in an initial consultation to inform them about the research methods and apply eligibility criteria.

### 2.4 Eligibility criteria

The inclusion and exclusion criteria are presented in Table 1.

**Table 1:** Eligibility criteria.

Inclusion criteria	Exclusion criteria
Diagnosis of functional constipation, according to Rome IV criteria (2016) ^[4,5]^	Presence of any organic cause for constipation
Children with age between 7 and 18 yrs old	Children with neurological and/ or cognitive deficits
Patients and/or their guardians who agree to and sign the ICF	Presence of skin lesions in the region of application of the electrodes
Patients aged 11 to 18 who agree and sign the respective SCF	An altered sensation at the electrode application site
Use of cardiac pacemaker	
Children with heart disease or cardiac arrhythmias	
Patients who do not complete the intervention or do not participate in some of the evaluation interviews	

### 2.5 Intervention

**Figure 1:**
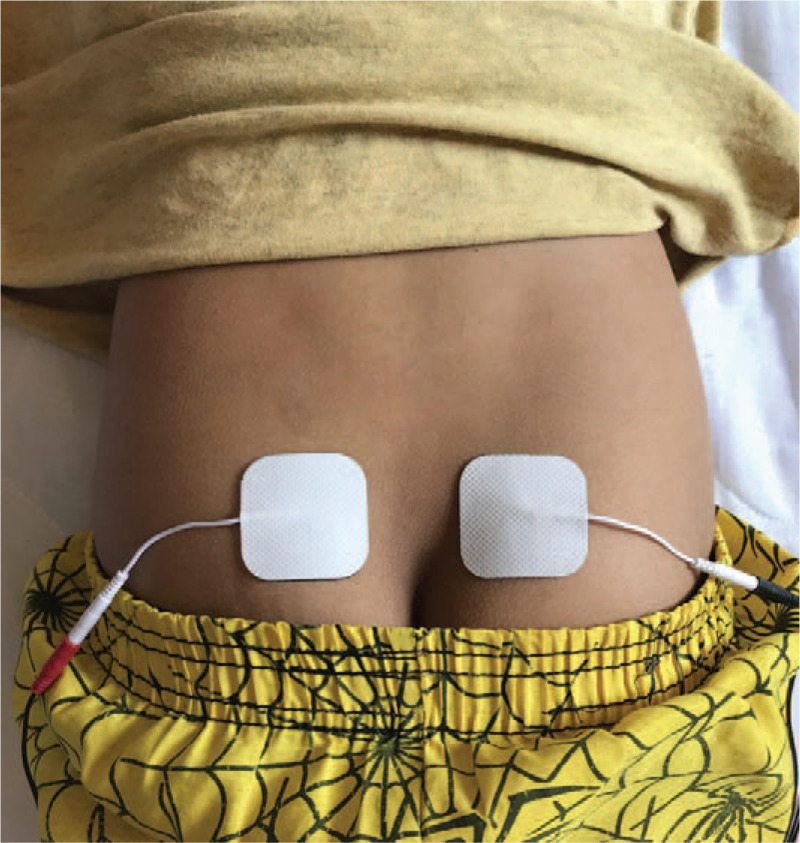
Electrode positioning for transcutaneous parasacral nerve stimulation.

Patients will be submitted to TPNS based on previously described methods.^[28,31]^ A physiotherapist, a member of the research team with experience in this therapy, will supervise all the procedures. Two silicone auto adhesive electrodes (5 × 5 cm) will be applied on each side of the sacrum at the level of S2 to S4 (Fig. 1). The electrodes will be linked to an electrical stimulation device (De Tens/Fes - 2 channels, Neurodyn Portable Ibramed). The maximum level tolerated by the child but without reaching the motor, the point will set the intensity level. A 500-μs, 10-Hz current will be applied 30 minutes daily for 4 or 8 consecutive weeks in the patient's residence.

Parents or guardians will be trained to perform TPNS on their children. Training courses and supervised applications will be provided until parents are confident enough to implement TPNS without supervision. From then on, TPNS will be executed daily in the family. During the intervention period, patients will need to attend a supervised session of TPNS every 15 days for a revaluation of the technique and clarification of any doubts.

During the intervention period, patients must maintain all the treatment recommendations (drugs, diets, and behaviors) previously prescribed by the referring physician. Any necessary modifications in treatment during this period will not be avoided or postponed because of the study. Patients must call the research team any changes to the treatment plan during the intervention.

### 2.6 Adherence to treatment and related adverse events

The biweekly-supervised TPNS sessions will be registered by the studies, also investigating the possibility of adverse events. During the research process, patients/parents/guardians can contact the research team by phone to communicate possible adverse events and clarify any questions.

### 2.7 Intervention periods and assessment time points

The intervention period will be 4 consecutive weeks, immediately following the conclusion of the training period. Then, patients and/or their guardians may choose to extend the intervention period for another 4 consecutive weeks until completing 8 weeks of TPNS. The 3 or 4 moments for assessment interviews will be the following: 1 week before the start of the intervention, immediately after the 4 weeks of intervention or immediately after the 4 and 8 weeks of intervention, and 4 weeks after the end of the intervention period, to identify any remaining effects of TPNS.

### 2.8 Assessment interviews

In all assessment moments, patients and/or their guardians will participate in semi-structured interviews to determine the results of interventions regarding bowel movements and quality of life. The same member of the research team will be responsible for the interviews that will last approximately 40 minutes. The following surveys will be applied: a questionnaire addressing the current clinical status developed by our research group and previously used in another study that evaluated the impact of transcutaneous posterior tibial nerve stimulation in children and adolescents with FC^[31]^ (see questionnaire, Supplemental Digital Content 4); the modified Bristol Stool Form Scale for children (mBSFS-C)^[32–34]^ to analyze the stool consistency (see mBSFS-C, Supplemental Digital Content 5); the Bowel Function Score (BF-S)^[35]^ to evaluate the functional status of bowel habits (seeBF-S, Supplemental Digital Content 6); the Fecal Continence Index (FCI) questionnaire,^[36,37]^ based on the clinical evaluation of fecal continence (see FCI, Supplemental Digital Content 7); the Assessment of Quality of Life in Children and Adolescents with Fecal Incontinence (AQLCAFI)^[36,37]^ (see AQLCAFI, Supplemental Digital Content 8) and the Pediatric Quality of Life Inventory version 4.0 (PEDsQL 4.0)^[38,39]^ to assess the quality of life (see PEDsQL 4.0, Supplemental Digital Content 9). Lastly, a questionnaire addressing the evaluation of the applicability of the TPNS daily home sessions^[31]^ also developed and already used by our research group (see applicability questionnaire, Supplemental Digital Content 10).

In addition, patients will be monitored using a standardized week diary (stool frequency, stool consistency according to the mBSFS-c, frequency of fecal incontinence episodes, painful defecation, abdominal pain, excretion of blood in the stool, laxative use and their doses, and adverse effects such as pain, nausea, vomiting, diarrhea, and flatulence). The fulfilment of this diary will start seven days before each assessment moment. The doses of laxative medications will be registered, addressing any alterations as possible outcomes in the study's conclusion.

### 2.9 Outcomes

Data analysis will focus on each assessment moment. The following clinical variables will be used for this analysis: the number of bowel movements per week, the number of fecal incontinence episodes per week, whether diapers or underwear protection is needed, whether defecation medications are needed, special diets, straining and pain while passing stool, presence of fecalomas, presence of abdominal pain, presence of bloody stools, need for enemas, and presence of perianal rashes or dermatitis. We will also analyze the results obtained by all the assessment instruments applied (mBSFS-C, BF-S, FCI questionnaire, AQLCAFI, and PEDsQL 4.0) to evaluate the outcomes of the functional status of bowel habits and the global quality of life.

**Figure 2:**
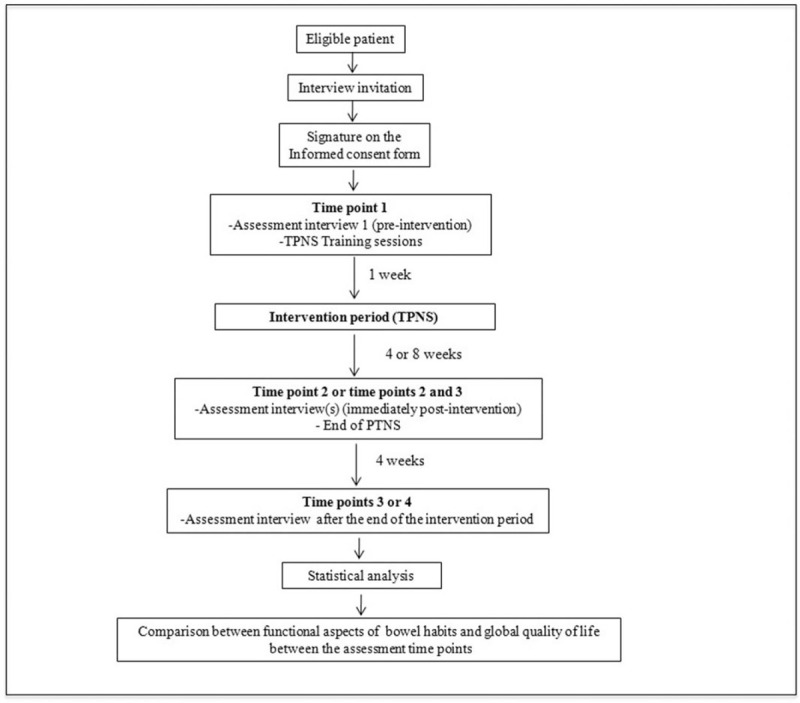
Flowchart of patients in the study.

As a secondary outcome, the applicability of TPNS will also be evaluated through the results of a specific questionnaire^[31]^ (see applicability questionnaire, Supplemental Digital Content 10), taking into account the practical difficulties and possible adverse events involved in using the method. All conditions that may be indirectly involved in the clinical results of TPNS, such as modifications in laxative doses or any clinical or dietary changes, will also be analyzed as secondary endpoints. Figure 2 summarizes the stages of the study and the Supplemental Digital Content 11 (SDC-11) features information about the Standard Protocol Items: Recommendations for Interventionist Tests (SPIRIT).

### 2.10 Statistical analysis

A descriptive analysis will be performed to characterize the patients before the beginning of the TPNS. A comparative statistical analysis will be conducted involving the results obtained for the variables analyzed at each evaluation time point. Continuous numerical data will be expressed as the mean ± standard deviation and median and Interquartile range (IQR). Proportions will be presented as percentages with their respective reliability intervals.

The comparison between the assessment time points will be performed using different statistical tests, according to the types of variables analyzed and to the normality distribution of data obtained by the results of the Shapiro-Wilk and Kolmogorov-Smirnov tests. Nominal variables will be analyzed using the McNemar test. Continuous numerical variables of parametric distribution will be analyzed using the *t*-test for paired samples. Continuous numerical variables of nonparametric distribution and ordinal variables will be analyzed using the Wilcoxon test. The significance level will be determined as 5% and will be analyzed using SPSS 22.0 software for Windows.

### 2.11 Protocol amendments

REC will approve any modifications in the study before implementation. Data and source documents will be stored so that they can be accessed later by the REC. At the end of the study, the results will be submitted for publication in a peer-reviewed journal, following CONSORT compliance guidelines. Authorship of any related presentations or reports will be under the name of the collaborative group.

## 3 Discussion

TPNS can be considered a promising, noninvasive, and safe method to be used as adjunctive therapy for treating constipation.^[18,27–30]^ However, there is a lack of published data that has investigated its use directed to treatment of children with FC.

This study's main objective was to assess the applicability and clinical outcomes of TPNS in children with FC. This study aims to demonstrate the efficacy of this promising method to increase the number of bowel movements and to improve stool consistency, to reduce the number of episodes of retentive fecal incontinence and to improve the overall quality of life indirectly.

Our research group has already developed a similar study to evaluate the applicability and clinical results of posterior tibial nerve stimulation in children with functional constipation.^[31]^ Thus, it will also be possible to compare the results obtained by these 2 minimally invasive neurostimulation methods in this clinical setting.

This study is currently in the planning phase, with the recruitment phase planned to begin in November 2020.

## Author contributions

**Conceptualization:** Pedro Luiz Toledo de Arruda Lourenção, Giovanna Maria Coelho, Nilton Carlos Machado, Mary de Assis Carvalho, Rebeca Mayara Padilha Rego, Stefani Rabelo Vieira, Erika Veruska Paiva Ortolan.

**Formal analysis:** Pedro Luiz Toledo de Arruda Lourenção, Nilton Carlos Machado.

**Funding acquisition:** Pedro Luiz Toledo de Arruda Lourenção, Erika Veruska Paiva Ortolan.

**Investigation:** Giovanna Maria Coelho, Nilton Carlos Machado, Mary de Assis Carvalho.

**Methodology:** Pedro Luiz Toledo de Arruda Lourenção, Giovanna Maria Coelho, Nilton Carlos Machado, Mary de Assis Carvalho, Rebeca Mayara Padilha Rego, Stefani Rabelo Vieira, Erika Veruska Paiva Ortolan.

**Project administration:** Pedro Luiz Toledo de Arruda Lourenção, Giovanna Maria Coelho, Nilton Carlos Machado.

**Supervision:** Pedro Luiz Toledo de Arruda Lourenção, Nilton Carlos Machado.

**Validation:** Mary de Assis Carvalho.

**Writing – original draft:** Pedro Luiz Toledo de Arruda Lourenção, Giovanna Maria Coelho, Rebeca Mayara Padilha Rego, Stefani Rabelo Vieira.

**Writing – review & editing:** Pedro Luiz Toledo de Arruda Lourenção, Giovanna Maria Coelho, Nilton Carlos Machado, Mary de Assis Carvalho, Erika Veruska Paiva Ortolan.

## Supplementary Material

Supplemental Digital Content

## Supplementary Material

Supplemental Digital Content

## Supplementary Material

Supplemental Digital Content

## Supplementary Material

Supplemental Digital Content

## Supplementary Material

Supplemental Digital Content

## Supplementary Material

Supplemental Digital Content

## Supplementary Material

Supplemental Digital Content

## Supplementary Material

Supplemental Digital Content

## Supplementary Material

Supplemental Digital Content

## Supplementary Material

Supplemental Digital Content

## Supplementary Material

Supplemental Digital Content
