# Supplementary material for: A protocol for an interventional study on the impact of transcutaneous parasacral nerve stimulation in children with functional constipation
Source: Medicine (Baltimore). 2020 Dec 18;99(51):e23745. doi: 10.1097/MD.0000000000023745 (PMC7748169; doi:10.1097/MD.0000000000023745)

## Slide 1
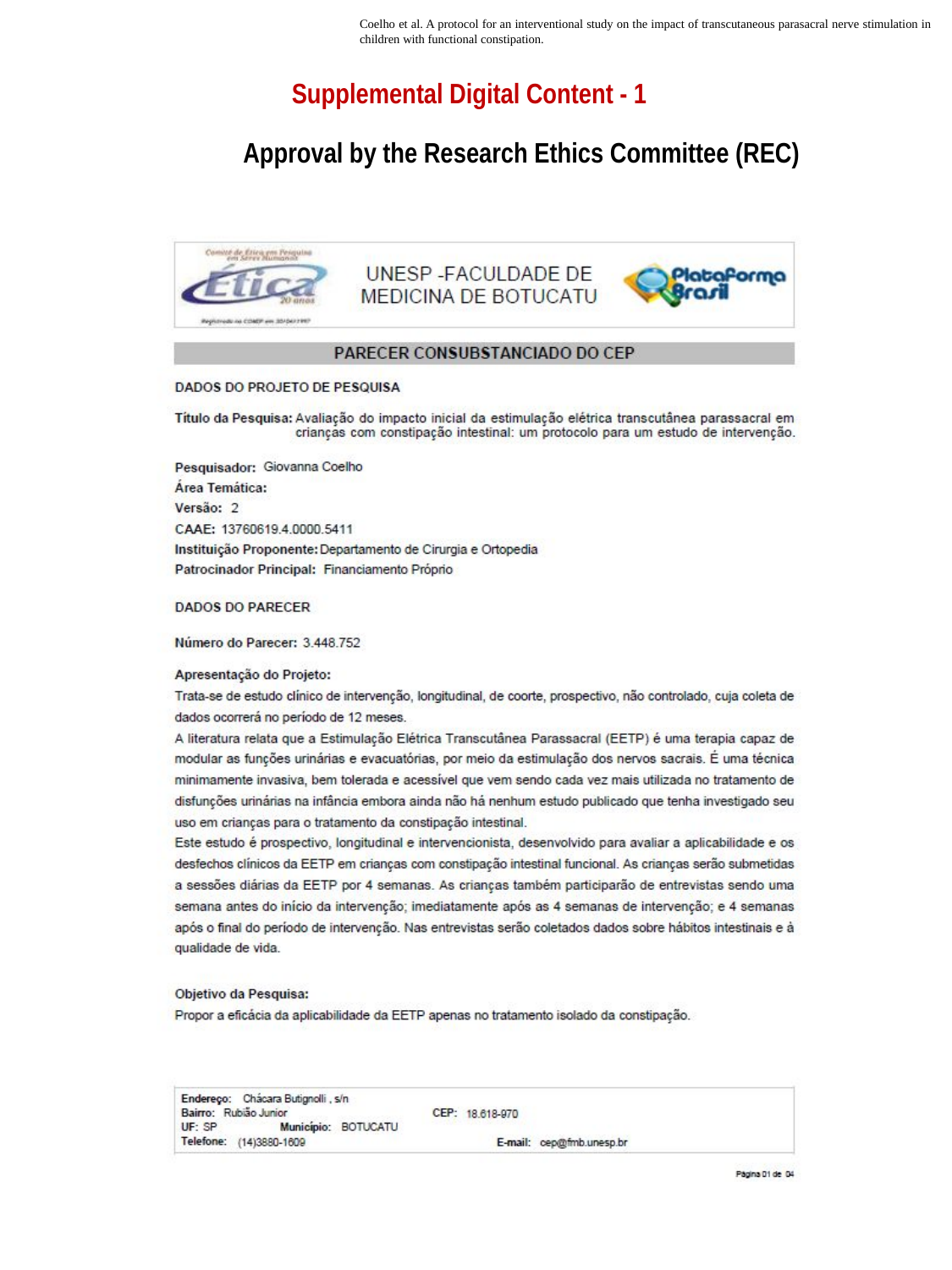

Coelho et al. A protocol for an interventional study on the impact of transcutaneous parasacral nerve stimulation in children with functional constipation.
Supplemental Digital Content - 1
Approval by the Research Ethics Committee (REC)

## Slide 2
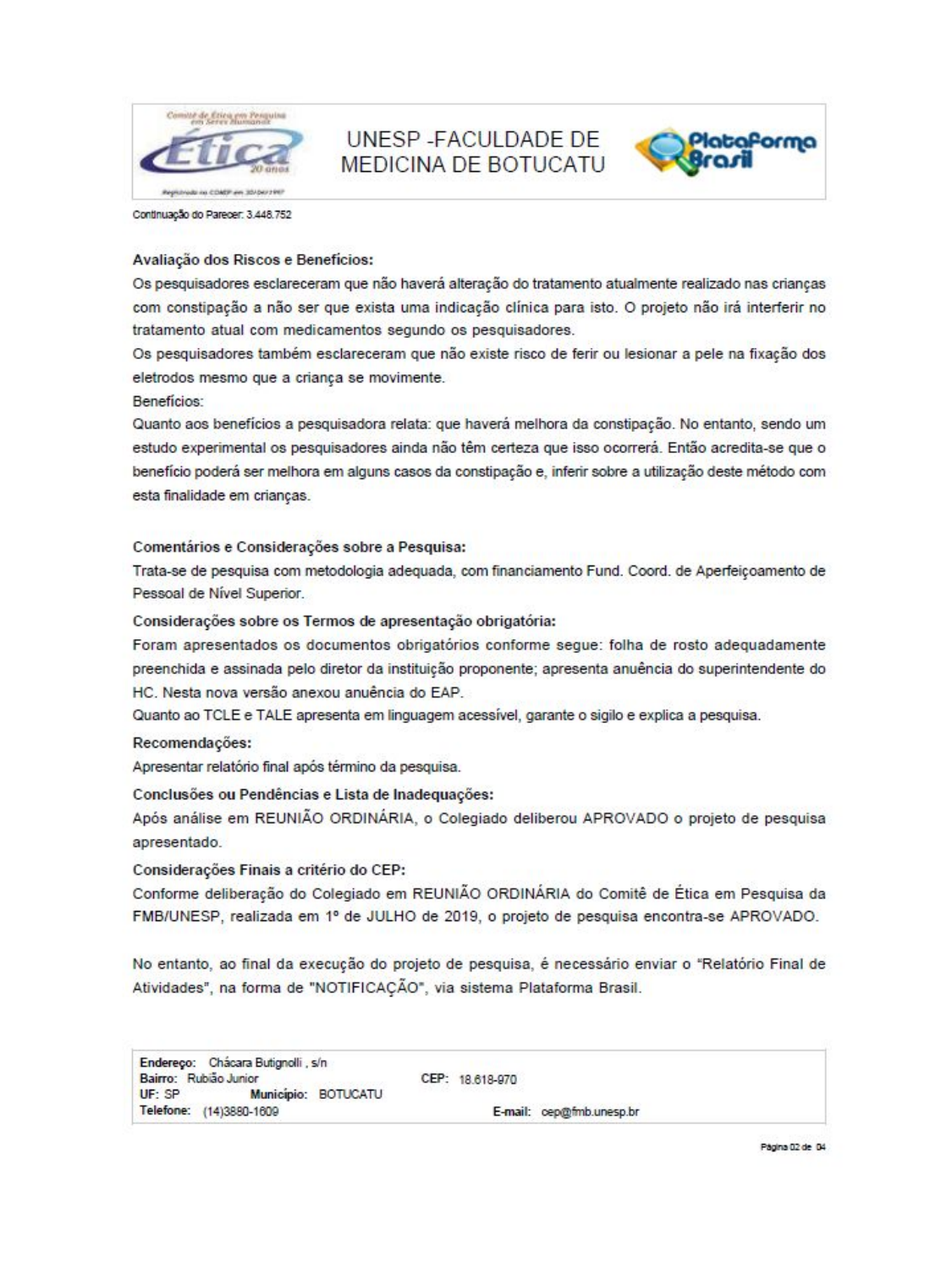

## Slide 3
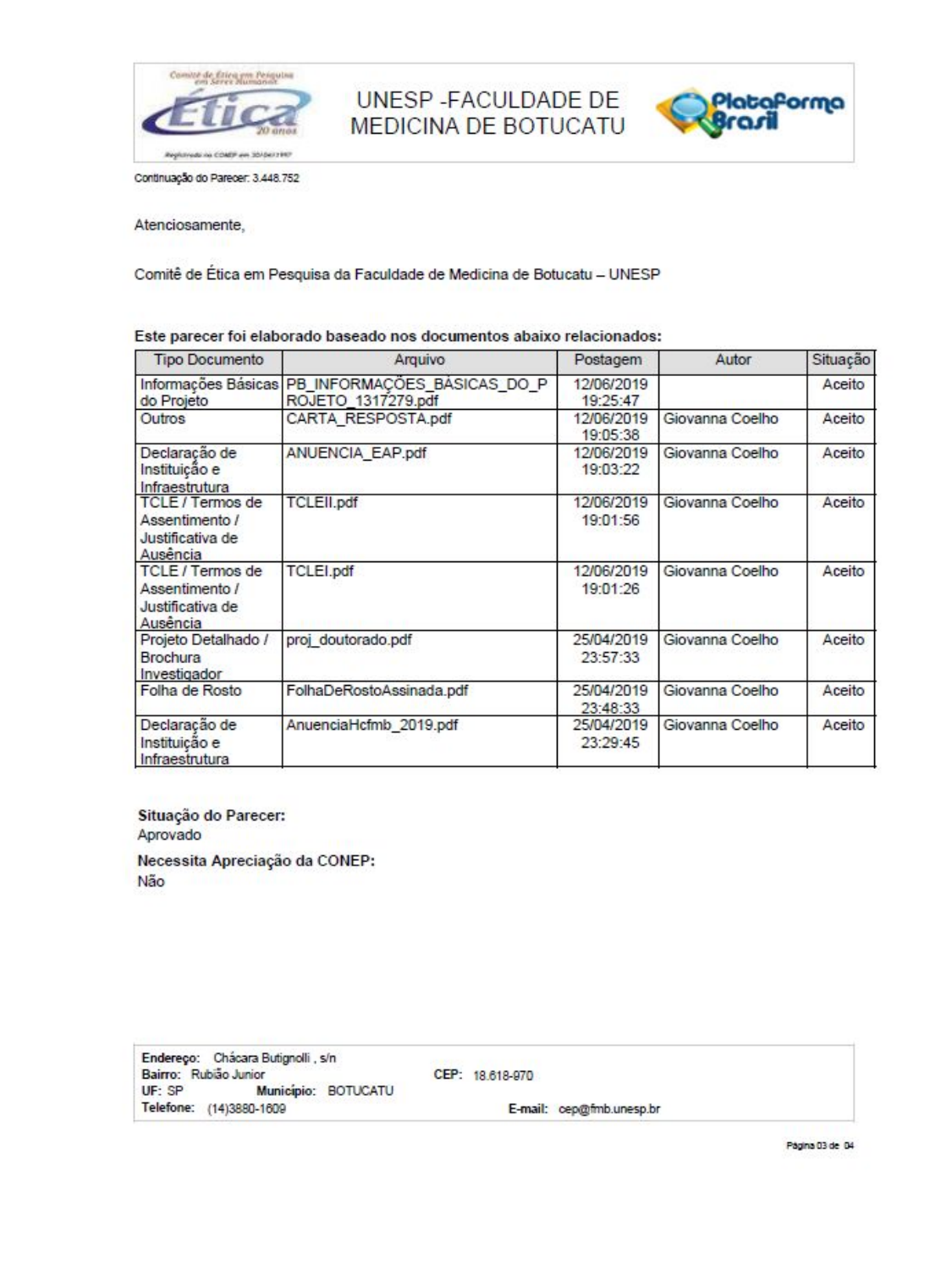

## Slide 4
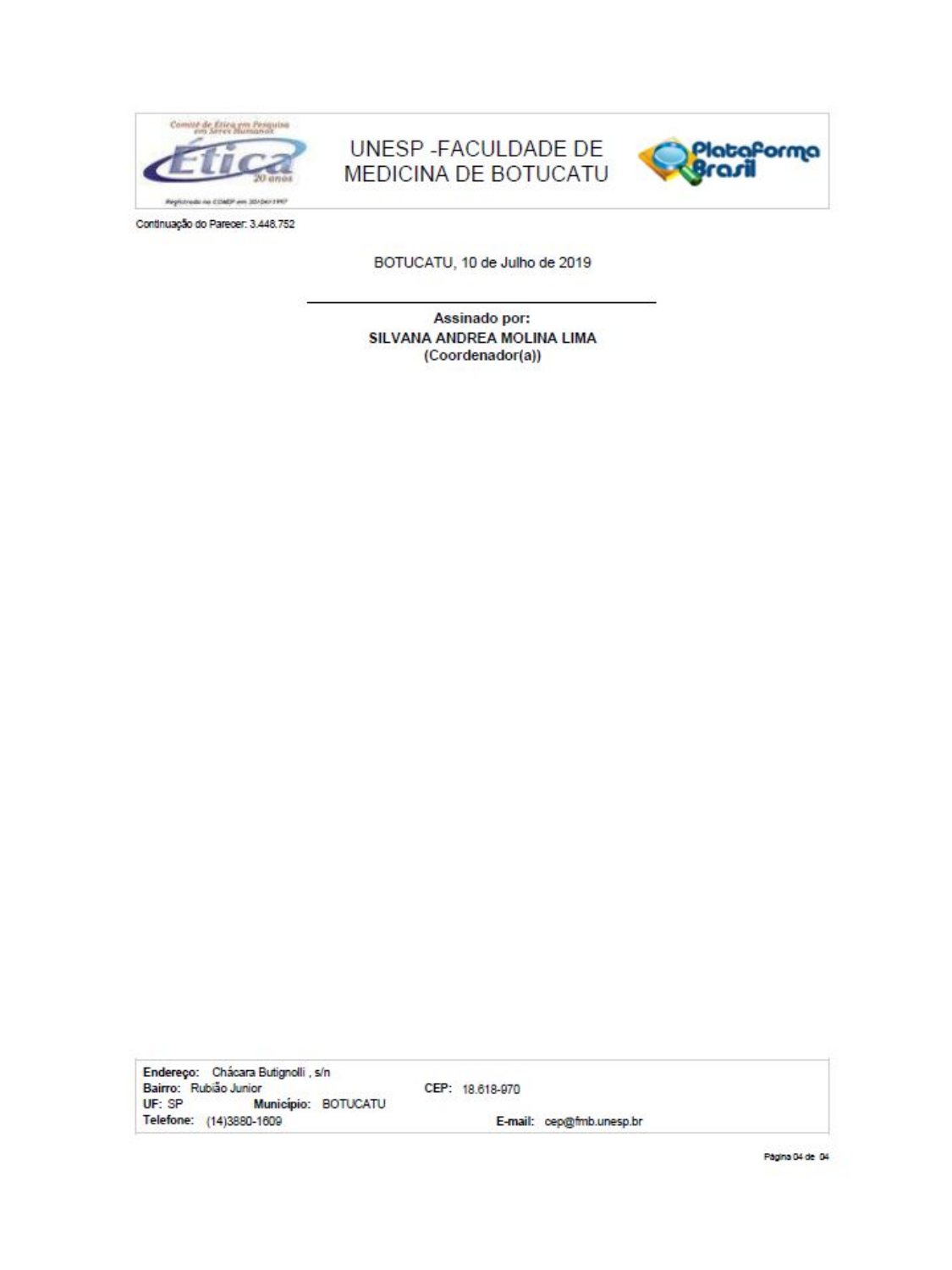

Supplement: Supplemental Digital Content [file medi-99-e23745-s001.pptx]
