# Supplementary material for: A protocol for an interventional study on the impact of transcutaneous parasacral nerve stimulation in children with functional constipation
Source: Medicine (Baltimore). 2020 Dec 18;99(51):e23745. doi: 10.1097/MD.0000000000023745 (PMC7748169; doi:10.1097/MD.0000000000023745)
Supplement: Supplemental Digital Content [file medi-99-e23745-s002.docx]

**Supplemental Digital Content - 2**

Coelho et al. A protocol for an interventional study on the impact of transcutaneous parasacral nerve stimulation in children with functional constipation.

**Informed Consent Form (ICF)**

**TERMO DE CONSENTIMENTO LIVRE E ESCLARECIDO**

CONVIDO, você,______________________________________ para participar do projeto de pesquisa intitulado “**Avaliação do impacto inicial da eletroestimulação transcutânea parassacral em crianças com constipação intestinal: um protocolo para um estudo de intervenção.**”, que será executado pela fisioterapeuta e aluna de doutorado Giovanna Maria Coelho com orientação do profissional médico e Professor (a) Dr. Pedro Luiz Toledo de Arruda Lourenção, da Faculdade de Medicina de Botucatu – UNESP.

Nós estamos investigando a utilização de uma nova terapia para crianças e adolescentes com constipação intestinal, como o seu caso. Esta nova terapia acontece pela estimulação nervosa de um nervo na região parassacral (região lombar), que consegue estimular o intestino a funcionar melhor. Alguns estudos já demonstraram que esse tratamento pode ser eficaz, mas nós decidimos estudá-lo para conhecer melhor os seus possíveis resultados.

Se você deseja participar do estudo, será preciso que você aplique em casa a eletroestimulação por 30 minutos, todos os dias, em um período de 4 semanas. Durante a estimulação você não sentirá dor alguma, mas pode ser que apresente uma sensação parecida como se tivessem várias “formiguinhas” passando de um lado para o outro para pelas suas costas. Antes do procedimento, você terá um treinado e só fará a eletroestimulação em casa quando se sentir seguro o suficiente.

Será preciso, também, que uma semana antes de toda avaliação marcada, você preencha um diário que vamos te fornecer, relacionado ao seu hábito intestinal. São informações sobre frequência em que foi no banheiro, forma e consistência do cocô e episódios de perdas fecais. Eu entrarei em contato por telefone para recordá-lo (a) sobre o preenchimento e você poderá entrar em contato com a equipe de pesquisa caso tenha alguma dúvida ou para relatar algum evento adverso.

O benefício que você terá em participar será a possibilidade de melhorar os sintomas de constipação intestinal, como por exemplo, o aumento da frequência evacuatória e a redução de dor ou dificuldade ao evacuar e melhora da qualidade de vida. Como se trata de uma pesquisa, não podemos garantir que este tratamento traga, com total certeza, esses benefícios. Ao termino do estudo, se você achar que o tratamento te tez bem e quiser continuar será mantido até completar 6 meses de duração, e após este período, o quadro clínico será novamente reavaliado.

Fique ciente que a sua participação no estudo é voluntária e que mesmo após ter dado seu consentimento para participar da pesquisa, você poderá se retirar a qualquer momento, sem qualquer prejuízo na continuidade do tratamento.

Este Termo de Consentimento Livre e Esclarecido será elaborado em duas vias de igual teor, o qual uma via será entregue para você devidamente rubricada, e a outra via será arquivada e mantida pelos pesquisadores por um período de 5 anos após o término da pesquisa.

Qualquer dúvida adicional você poderá entrar em contrato com o Comitê de Ética em Pesquisa através dos telefones (14) 3880-1608 ou 3880-1609 que funciona de 2ª a 6ª feira das 8.00 às 11.30 e das 14.00 às 17horas, na Chácara Butignolli s/nº em Rubião Júnior – Botucatu - São Paulo. Os dados de localização dos pesquisadores estão abaixo descrito:

Após terem sido sanadas todas minhas dúvidas a respeito deste estudo, CONCORDO em participar de forma voluntária, estando ciente que todos os seus dados estarão resguardos através do sigilo que os pesquisadores se comprometeram. Estou ciente que os resultados desse estudo poderão ser publicados em revistas científicas, sem, no entanto, que a identidade minha seja revelada.

Botucatu,_____/___/______

_____________________ _________________________

Pesquisador Participante da Pesquisa

Giovanna Maria Coelho

Endereço: Departamento de Cirurgia e Ortopedia – Anexo Verde – 3º andar. Faculdade de Medicina de Botucatu – UNESP. Av. Prof. Mário Rubens Guimarães Montenegro, s/n. Bairro: UNESP - Campus de Botucatu. CEP: 18618-687 - Botucatu, SP Telefone: (14) 3880-1703 Email: [giovannamcoelho@hotmail.com](mailto:giovannamcoelho@hotmail.com)

Orientador: Prof. Dr. Pedro Luiz Toledo de Arruda Lourenção

Endereço: Departamento de Cirurgia e Ortopedia – Anexo Verde – 3º andar. Faculdade de Medicina de Botucatu – UNESP. Av. Prof. Mário Rubens Guimarães Montenegro, s/n. Bairro: UNESP - Campus de Botucatu. CEP: 18618-687 - Botucatu, SP Telefone: (14) 3880-1703 Email: lourencao@fmb.unesp.br
