# Supplementary material for: A protocol for an interventional study on the impact of transcutaneous parasacral nerve stimulation in children with functional constipation
Source: Medicine (Baltimore). 2020 Dec 18;99(51):e23745. doi: 10.1097/MD.0000000000023745 (PMC7748169; doi:10.1097/MD.0000000000023745)
Supplement: Supplemental Digital Content [file medi-99-e23745-s003.docx]

**Supplemental Digital Content - 3**

**Specific Consent Form (SCF)**

Coelho et al. A protocol for an interventional study on the impact of transcutaneous parasacral nerve stimulation in children with functional constipation.

**TERMO DE ASSENTIMENTO LIVRE E ESCLARECIDO (TALE)**

CONVIDO, o (a) Senhor (a),_____________________________________ responsável pelo(a) menor _________________________________________________ para participar do Projeto de Pesquisa intitulado “**Avaliação do impacto inicial da eletroestimulação transcutânea parassacral em crianças com constipação intestinal: um protocolo para um estudo de intervenção.**”, que será executado pela fisioterapeuta e aluna de doutorado Giovanna Maria Coelho com orientação do profissional médico e Professor (a) Dr. Pedro Luiz Toledo de Arruda Lourenção, da Faculdade de Medicina de Botucatu – UNESP.

Nós estamos investigando a utilização de uma nova terapia para crianças e adolescentes com constipação intestinal, como no caso do seu (ua) filho (a). Esta terapia acontece pela estimulação nervosa de um nervo na região lombar, que consegue estimular o intestino a funcionar melhor. Alguns estudos já demonstraram que esse tratamento pode ser eficaz, mas nós decidimos estudá-lo para conhecer melhor os seus possíveis resultados.

Se você deseja que seu (ua) filho (a) participe do estudo, será preciso que você junto a ele (a) aplique em casa a eletroestimulação por 30 minutos, todos os dias, em um período de 4 semanas. Durante a estimulação seu (ua) filho (a) não sentirá dor alguma, mas pode ser que sinta uma sensação parecida como se tivessem várias “formiguinhas” passando de um lado para o outro nas costas. Antes de realizar o procedimento em casa, você responsável, terá um treinamento e só irão iniciar a eletroestimulação quando se sentirem seguros para isso.

Será preciso, também, que uma semana antes de toda avaliação marcada, você preencha um diário que vamos te fornecer, relacionado ao seu hábito intestinal dele (a). São informações sobre o número de vezes que estará fazendo cocô, a forma e a consistência do mesmo e episódios de perdas fecais. Eu entrarei em contato por telefone para recordá-lo (a) sobre o preenchimento e você poderá entrar em contato com a equipe de pesquisa caso tenha alguma dúvida ou para relatar algum evento adverso.

O benefício que seu filho (a) terá em participar será a possibilidade de melhorar os seus sintomas de constipação intestinal, como por exemplo, o aumento do número de vezes que você faz cocô e a diminuição da dor e do esforço para evacuar. Como se trata de uma pesquisa, não podemos garantir que este tratamento traga, com total certeza, esses benefícios. Ao termino do estudo, se você e seu(ua) filho(a) responsável acharem que o tratamento está fazendo e quiserem continuar, o tratamento será mantido até completar 6 meses de duração, e após este período, o quadro clínico será novamente reavaliado.

Fique ciente, que a participação neste estudo é voluntária e que mesmo após ter dado consentimento para participar da pesquisa, você poderá retirar a qualquer momento seu filho (a), sem qualquer prejuízo na continuidade do tratamento, ou qualquer outra atividade.

Este Termo de Assentimento Livre e Esclarecido será elaborado em duas vias de igual teor, o qual uma via será entregue a você devidamente rubricada, e a outra via será arquivada e mantida pelos pesquisadores por um período de 5 anos após o término da pesquisa.

Qualquer dúvida adicional você poderá entrar em contrato com o Comitê de Ética em Pesquisa através dos telefones (14) 3880-1608 ou 3880-1609 que funciona de 2ª a 6ª feira das 8.00 às 11.30 e das 14.00 às 17horas, na Chácara Butignolli s/nº em Rubião Júnior – Botucatu - São Paulo.

Após terem sido sanadas todas minhas dúvidas a respeito deste estudo, CONCORDO que meu filho (a) participe de forma voluntária, estando ciente que todos os meus dados estarão resguardos através do sigilo que os pesquisadores se comprometeram. Estou ciente que os resultados desse estudo poderão ser publicados e revistas científicas, sem que a minha identidade seja revelada.

Botucatu,_____/___/______

_______________________ _________________________

Pesquisador Participante da Pesquisa

Giovanna Maria Coelho

Endereço: Departamento de Cirurgia e Ortopedia – Anexo Verde – 3º andar. Faculdade de Medicina de Botucatu – UNESP. Av. Prof. Mário Rubens Guimarães Montenegro, s/n. Bairro: UNESP - Campus de Botucatu. CEP: 18618-687 - Botucatu, SP Telefone: (14) 3880-1703 Email: giovannamcoelho@hotmail.com

Orientador: Prof. Dr. Pedro Luiz Toledo de Arruda Lourenção

Endereço: Departamento de Cirurgia e Ortopedia – Anexo Verde – 3º andar. Faculdade de Medicina de Botucatu – UNESP. Av. Prof. Mário Rubens Guimarães Montenegro, s/n. Bairro: UNESP - Campus de Botucatu. CEP: 18618-687 - Botucatu, SP Telefone: (14) 3880-1703 Email: lourencao@fmb.unesp.br
