# Supplementary material for: A protocol for an interventional study on the impact of transcutaneous parasacral nerve stimulation in children with functional constipation
Source: Medicine (Baltimore). 2020 Dec 18;99(51):e23745. doi: 10.1097/MD.0000000000023745 (PMC7748169; doi:10.1097/MD.0000000000023745)
Supplement: Supplemental Digital Content [file medi-99-e23745-s004.pptx]

## Slide 1
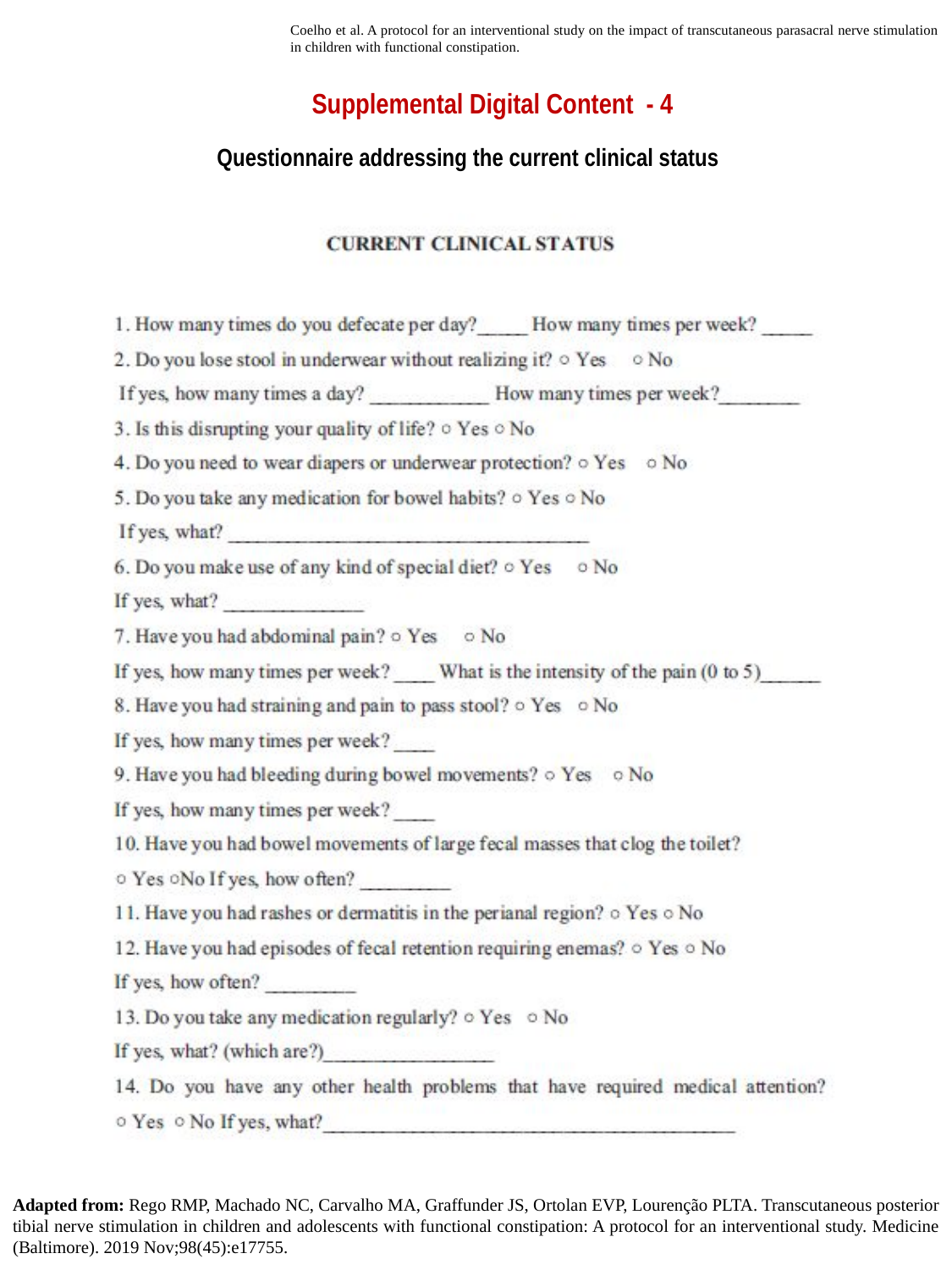

Coelho et al. A protocol for an interventional study on the impact of transcutaneous parasacral nerve stimulation in children with functional constipation.
Supplemental Digital Content - 4
Questionnaire addressing the current clinical status
Adapted from: Rego RMP, Machado NC, Carvalho MA, Graffunder JS, Ortolan EVP, Lourenção PLTA. Transcutaneous posterior tibial nerve stimulation in children and adolescents with functional constipation: A protocol for an interventional study. Medicine (Baltimore). 2019 Nov;98(45):e17755.
