# Supplementary material for: A protocol for an interventional study on the impact of transcutaneous parasacral nerve stimulation in children with functional constipation
Source: Medicine (Baltimore). 2020 Dec 18;99(51):e23745. doi: 10.1097/MD.0000000000023745 (PMC7748169; doi:10.1097/MD.0000000000023745)
Supplement: Supplemental Digital Content [file medi-99-e23745-s005.pptx]

## Slide 1
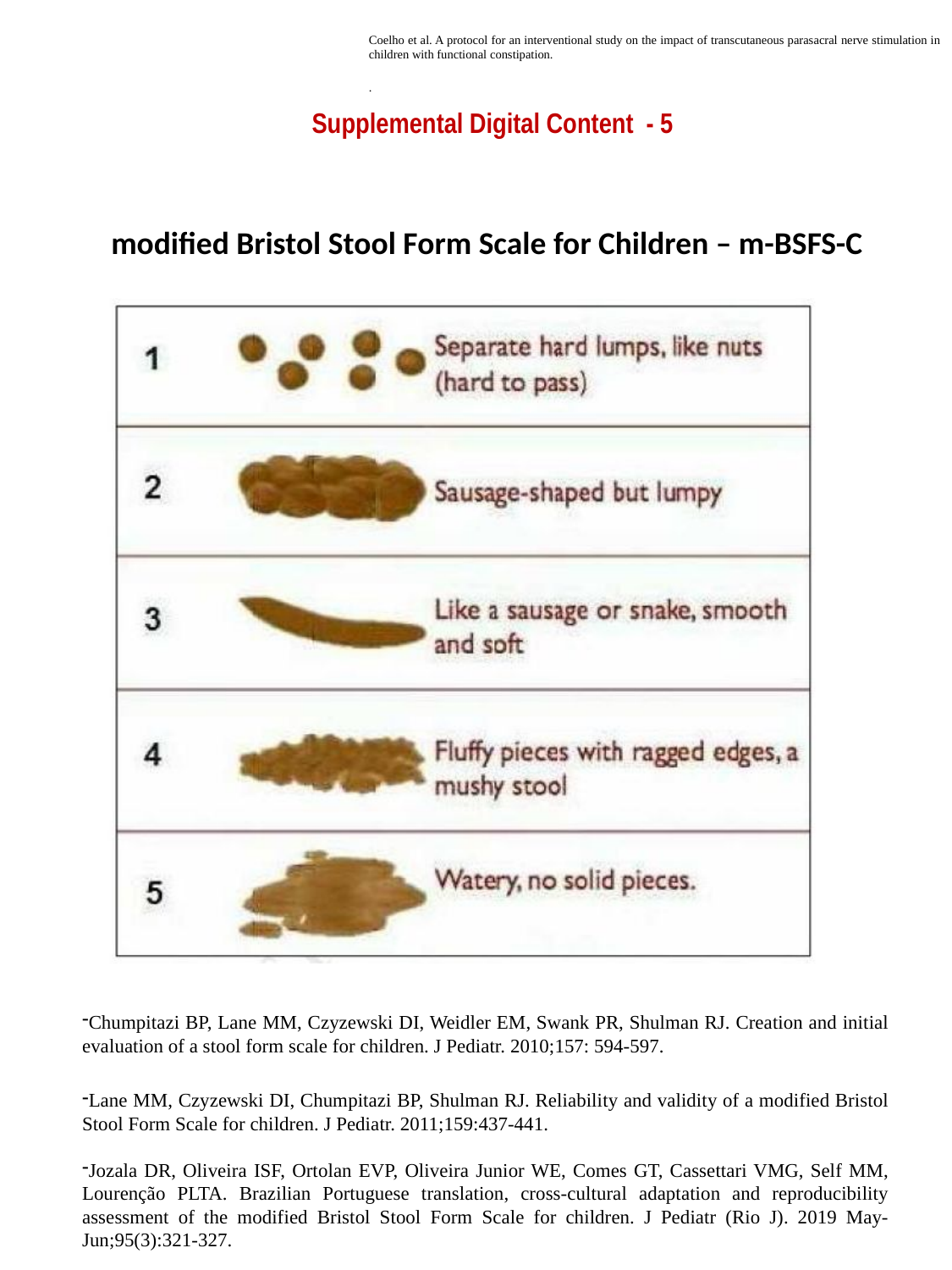

Coelho et al. A protocol for an interventional study on the impact of transcutaneous parasacral nerve stimulation in children with functional constipation.
.
Supplemental Digital Content - 5
modified Bristol Stool Form Scale for Children – m-BSFS-C
Chumpitazi BP, Lane MM, Czyzewski DI, Weidler EM, Swank PR, Shulman RJ. Creation and initial evaluation of a stool form scale for children. J Pediatr. 2010;157: 594-597.
Lane MM, Czyzewski DI, Chumpitazi BP, Shulman RJ. Reliability and validity of a modified Bristol Stool Form Scale for children. J Pediatr. 2011;159:437-441.
Jozala DR, Oliveira ISF, Ortolan EVP, Oliveira Junior WE, Comes GT, Cassettari VMG, Self MM, Lourenção PLTA. Brazilian Portuguese translation, cross-cultural adaptation and reproducibility assessment of the modified Bristol Stool Form Scale for children. J Pediatr (Rio J). 2019 May-Jun;95(3):321-327.
