# Supplementary material for: A protocol for an interventional study on the impact of transcutaneous parasacral nerve stimulation in children with functional constipation
Source: Medicine (Baltimore). 2020 Dec 18;99(51):e23745. doi: 10.1097/MD.0000000000023745 (PMC7748169; doi:10.1097/MD.0000000000023745)
Supplement: Supplemental Digital Content [file medi-99-e23745-s006.pptx]

## Slide 1
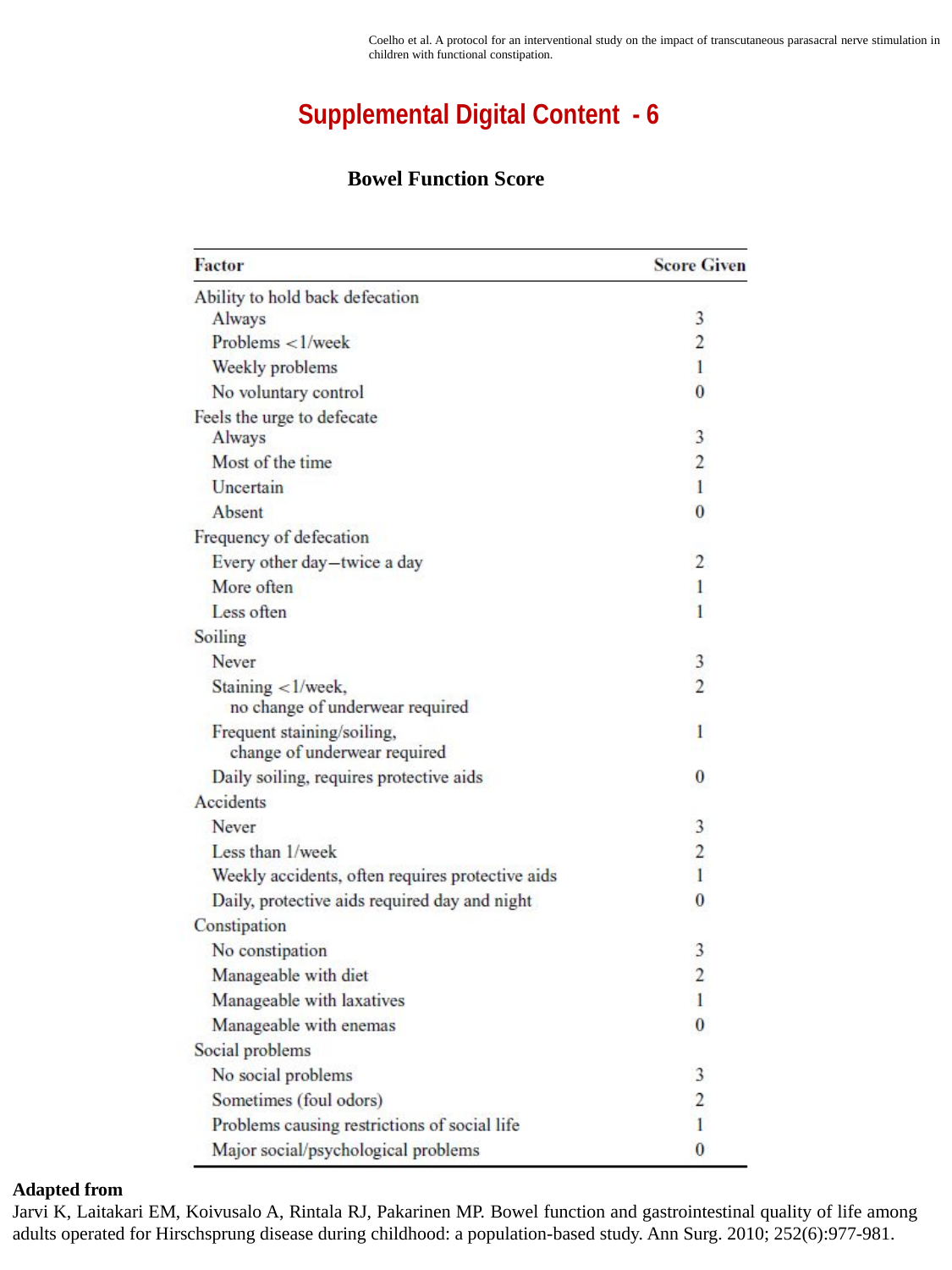

Coelho et al. A protocol for an interventional study on the impact of transcutaneous parasacral nerve stimulation in children with functional constipation.
Supplemental Digital Content - 6
Bowel Function Score
Adapted from
Jarvi K, Laitakari EM, Koivusalo A, Rintala RJ, Pakarinen MP. Bowel function and gastrointestinal quality of life among adults operated for Hirschsprung disease during childhood: a population-based study. Ann Surg. 2010; 252(6):977-981.
