# Supplementary material for: A protocol for an interventional study on the impact of transcutaneous parasacral nerve stimulation in children with functional constipation
Source: Medicine (Baltimore). 2020 Dec 18;99(51):e23745. doi: 10.1097/MD.0000000000023745 (PMC7748169; doi:10.1097/MD.0000000000023745)
Supplement: Supplemental Digital Content [file medi-99-e23745-s008.pptx]

## Slide 1
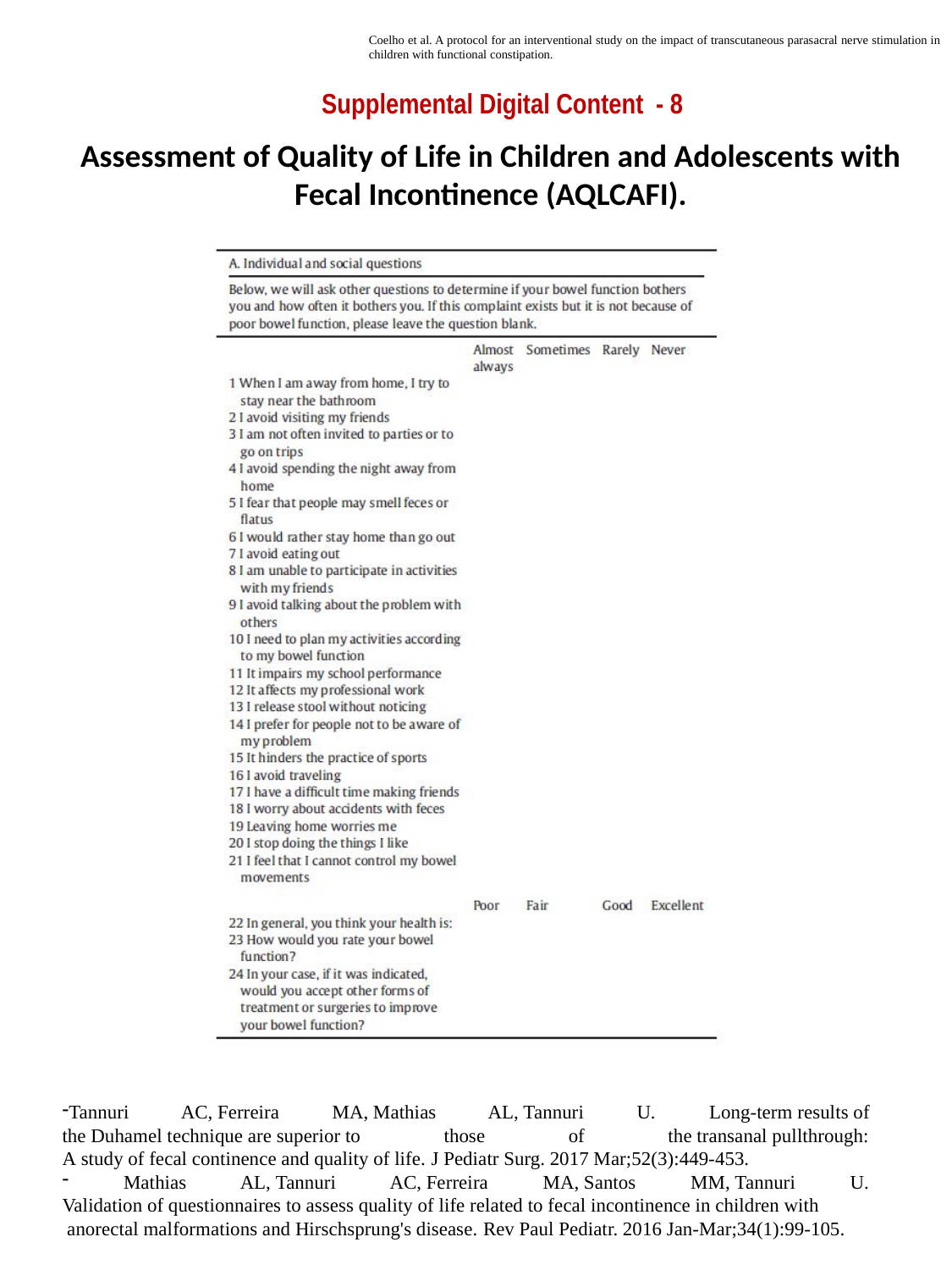

Coelho et al. A protocol for an interventional study on the impact of transcutaneous parasacral nerve stimulation in children with functional constipation.
Supplemental Digital Content - 8
Assessment of Quality of Life in Children and Adolescents with Fecal Incontinence (AQLCAFI).
Tannuri AC, Ferreira MA, Mathias AL, Tannuri U. Long-term results of the Duhamel technique are superior to those of the transanal pullthrough: A study of fecal continence and quality of life. J Pediatr Surg. 2017 Mar;52(3):449-453.
 Mathias AL, Tannuri AC, Ferreira MA, Santos MM, Tannuri U. Validation of questionnaires to assess quality of life related to fecal incontinence in children with  anorectal malformations and Hirschsprung's disease. Rev Paul Pediatr. 2016 Jan-Mar;34(1):99-105.
